# Deep brain stimulation for Tourette’s syndrome

**DOI:** 10.1186/s40035-020-0183-7

**Published:** 2020-01-13

**Authors:** Wenying Xu, Chencheng Zhang, Wissam Deeb, Bhavana Patel, Yiwen Wu, Valerie Voon, Michael S. Okun, Bomin Sun

**Affiliations:** 1 Department of Functional Neurosurgery, Ruijin Hospital, Shanghai Jiao Tong University School of Medicine, Ruijin Hospital, 197 Ruijin Er Road, Shanghai, 200025 China; 2 Norman Fixel Institute for Neurological Diseases, Department of Neurology, College of Medicine, University of Florida, Gainesville, FL 32608 USA; 3 Department of Neurology & Institute of Neurology, Ruijin Hospital, Shanghai Jiao Tong University School of Medicine, Shanghai, China; 4 Department of Psychiatry, University of Cambridge, Cambridge, UK

**Keywords:** Tourette syndrome, Deep brain stimulation, Capsulotomy, Adaptive close loop, Connectivity

## Abstract

Tourette syndrome (TS) is a childhood-onset neuropsychiatric disorder characterized by the presence of multiple motor and vocal tics. TS usually co-occurs with one or multiple psychiatric disorders. Although behavioral and pharmacological treatments for TS are available, some patients do not respond to the available treatments. For these patients, TS is a severe, chronic, and disabling disorder. In recent years, deep brain stimulation (DBS) of basal ganglia-thalamocortical networks has emerged as a promising intervention for refractory TS with or without psychiatric comorbidities. Three major challenges need to be addressed to move the field of DBS treatment for TS forward: (1) patient and DBS target selection, (2) ethical concerns with treating pediatric patients, and (3) DBS treatment optimization and improvement of individual patient outcomes (motor and phonic tics, as well as functioning and quality of life). The Tourette Association of America and the American Academy of Neurology have recently released their recommendations regarding surgical treatment for refractory TS. Here, we describe the challenges, advancements, and promises of the use of DBS in the treatment of TS. We summarize the results of clinical studies and discuss the ethical issues involved in treating pediatric patients. Our aim is to provide a better understanding of the feasibility, safety, selection process, and clinical effectiveness of DBS treatment for select cases of severe and medically intractable TS.

## Background

Tourette syndrome (TS) is a relatively common neuropsychiatric disorder characterized by sudden, rapid, repetitive, non-rhythmic, and stereotyped movements and/or vocalizations. Diagnosis of TS requires the presence of both multiple motor tics and at least one phonic tic, with a childhood-onset and a duration of more than one year [1]. The prevalence of TS is 0.3–0.8% in children [2, 3]. Tics usually emerge around age 7, and may wax and wane in frequency [4]. TS is frequently complicated by the presence of one or more comorbid psychiatric disorders, particularly attention deficit hyperactivity disorder (ADHD), obsessive-compulsive disorder (OCD), impulse control disorder (ICD), and/or a mood disorder [5–7]. Severity of symptoms vary among patients and for many, tics gradually become less severe during adolescence and most of them disappear in early adulthood [8]. Current treatments for TS mainly involve behavioral interventions and pharmacotherapy, especially α2 adrenergic agonists, dopamine antagonists, dopamine depleters, benzodiazepines, antiepileptic drugs, and botulinum toxin injections [9–12]. However, for some patients’ TS is a severe and chronic disorder that does not respond to conventional pharmacological or behavioral treatments. Moreover, some of these patients develop what has been coined by some experts as “malignant TS” which can result in hospitalizations and/or self-injurious behaviors (e.g., cervical myelopathy, bone fractures, retinal detachment). Some patients with malignant TS may experience temporary or permanent disabilities [13–16]. Neurosurgical intervention, such as deep brain stimulation can be used to optimize care of selected individuals with malignant TS [17].

Recently, both DBS and ablative neurosurgical procedures have been utilized in an effort to manage refractory symptoms in TS patients [18–21]. In contrast to DBS, ablative surgery is not reversible and uncertainty exists whether ablative techniques work better in terms of clinical effectiveness and adverse event-profile. For example, bilateral thalamic lesions have been associated with impaired speech, swallowing difficulties, and cognitive deficits [22]. Moreover, substantial clinical evidence exists for the effectiveness of DBS in hyperkinetic movement disorders, such as tremor, tardive dyskinesia, and chorea [23–25]. It is believed that the pathophysiology of TS is closely linked to the dysfunction of cortico-striato-pallido-thalamo-cortical networksand that the modulation of these networks could alleviate the clinical symptoms of TS [26, 27]. Large inter-individual differences have been observed in the clinical symptoms of TS, along with the type and severity of psychiatric comorbidities, and clinical response to DBS. In order to move the field of DBS treatment for TS forward and to improve individual patient outcomes, at least three major challenges need to be addressed: [1] patient and target selection, [2] ethical issues involved in treating pediatric patients, and [3] optimization of DBS, such as motor and vocal tics, mental health, daily functioning, and quality of life. In this article, we outline the challenges, progress, and promises of DBS treatment for TS. We evaluate the results of clinical studies and discuss several methodological and ethical issues involved in DBS treatment of pediatric patients. The aim of this review is to provide a better understanding of the feasibility, safety, and effectiveness of DBS treatment for carefully selected cases of severe and intractable TS.

## Main text

### Surgical treatment

In this review, we consider TS in terms of the diagnostic criteria of the DSM-V. The main classifier is the presence of tics, which can be categorized as motor and phonic tics, and further divided into simple and complex tics. Simple motor tics can affect any body part, but they commonly appear in the face, such as eye blinking, raising the eyebrows, head jerking, or tongue protrusion. Some patients also manifest complex motor tics, such as grimacing, echopraxia (imitating others’ movements), copropraxia (e.g., performing socially inappropriate gestures or inappropriate touching) or, in rare cases, self-injurious behavior (e.g., self-hitting, self-biting, pounding on objects). Additionally, the diagnostic criteria for TS requires that the patient presents with or has a history of phonic tic(s). Common simple phonic tics include sniffing, throat clearing, coughing, yawning, or making other simple meaningless sounds. Complex phonic tics include echolalia (repeating others’ words or phrases), coprolalia (yelling out socially inappropriate words or phrases) or verbigeration (repeating a word rapidly and involuntarily) [28].

Most individuals diagnosed with TS present with one or more comorbid psychiatric disorders. In one cross-sectional study of 1374 TS participants, approximately 86% met diagnostic criteria for one or more psychiatric comorbidities [29]. The psychiatric disorders that most commonly co-occur with TS are ADHD, OCD, sleep disorders, anxiety disorders, and depressive disorders [30]. Psychiatric comorbidities remain an issue of concern in DBS treatment for TS because the symptoms can be severe, chronic, and may have a greater impact on the patient’s functioning and quality of life compared to motor and phonic tics. The Revised 2006 Guideline of the Tourette Association of America (TAA) Database/Registry Group recommends the following prior to initiating DBS, 1) the patient’s psychiatric comorbidities be stabilized and 2) no active suicide or homicidal ideation for six months [17].

**Fig. 1:**
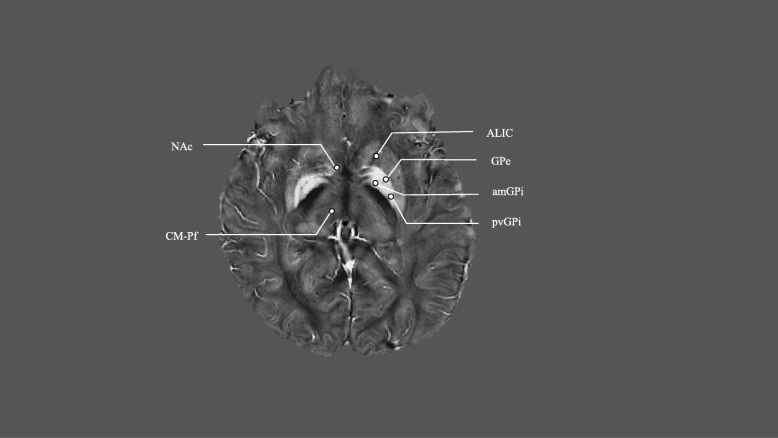
Quantitative susceptibility map of targets proposed for DBS in Tourette’s syndrome Abbreviations: ALIC, anterior limb of internal capsule; amGPi, anteromedial or limbic GPi; CM-Pf, centromedian-parafascicular complex; GPe, Globus Pallidus externus; NAc, Nucleus Accumbens; pvGPi, posteroventral GPi.

Clinician-researchers have collectively gravitated to a disease-centered approach to DBS for TS. This includes meticulous attention to patient selection [17], collection and analysis of standardized data [31], and engagement of multiple centers to identify patterns of symptoms and to improve outcomes [4]. Additionally, much effort has been put into identifying appropriate targets for DBS. Data from the TAA’s DBS Registry and Database have indicated that many different regions/structures located within the cortico-striato-pallido-thalamo-cortical network are promising targets [32].. In the next sections, we discuss some of the most promising DBS targets for TS treatment. Table 1 and Fig. 1 provide an overview of recent DBS treatment studies of patients with severe and refractory TS. The list is meant not to be exhaustive but rather to illustrate some of the current approaches in the field.

**Table 1:** Summary of the studies in this review

Study	Target	Patients (n)	Age of surgery (years)	Subjects (ages)	Follow-up	Study Design		Outcome of Tics	Outcome of Comorbidities and Quality of life	Side effects
				< 18	< 25					
Ladan et al.2017 [32]	amGPi	15	18–49	NR	NR	17–82 mo	Retrospective review	1. YGTSS 38.2% improvement motor scores 33.2% improvement vocal scores 38.2% improvement 2. 2.MRVRS 3. observed 40.5% improvement unobserved 34.1% improvement	1.OCB (Y-BOCS) (*n* = 15) severe OCB(*n* = 4) 38.7% improvement moderate OCB(*n* = 5) 12.3% improvement mild or subclinical OCB little or no improvement 2.Anxiety (STAI) significantly improve 3.Deprresion (BDI) significantly improve 4.GTS-QOL significantly improve	1.Stimulation-related weight gain, dizziness, feelings of nausea, freezing of gait episode, speech articulation, and akathisia
Rubens et al. 2016 [33]	CM-Pf	1	23	NR	1	18 mo	Case report	1.YGTSS 70.5% improvement impairment 60% improvement	1.Anxiety (HAS) 53% improvement	NR
Paola et al. 2016 [34]	CM-Pf	11	17–46	2	4	2–91 mo	Retrospective review	1.YGTSS 54% improvement motor scores 46% improvement vocal scores 52% improvement impairment 59% improvement	1.be employed(*n* = 7), enjoy an improve social life(n = 5), drive(*n* = 3) and go to college(n = 1)	1.Surgery-related: scalp erosion and purulent drainage 2.Postsurgical adverse effects: decreased memory, attention and mental flexibility, shock-like sensations, neck tightness, temporary anterograde amnesia, recurrent headache, nausea, vomiting, photophobia and phonophobia 3.Stimulation-induced: recurrent tension headache, worsening of pre-existing tremor, transient blurring of vision; intensity increase result dizziness and paresthesias; intensity decrease result dysarthria, involuntary movements of the tongue and jaw, and mouth opening, single seizure-like episode
Takanobu et al. 2010 [35]	CM-Pf-Voi	5	19–21	0	3	12 mo	Prospective, open-labeled case series study	1.YGTSS 52–71% improvement	1.OCD (Y-BOCS) improvement(*n* = 2) or exacerbation(n = 1) 2.Depression (BDI-II) improvement(*n* = 1) or exacerbation(n = 1) 3.Intelligence level full scale intelligence quotient score, FIQ from 64 to 82(n = 1) Performance intelligent quotient scores, PIQ from 78 ± 14 to 88 ± 13(n = 3) 4.Social impairment scores from 52 to 71% to 56–71%	1.Stimulation-related sensations of irritation, hotness of the body and blurred vision
Marano et al. 2019 [36]	CM-Pf	1	28	0	0	24 mo	Case report	1.YGTSS: sustained motor and phonic tic relief	1.OCB (SCL90), depression (BDI), and anxiety (BAI) showed a remarkable improvement	1.Stimulation-related: mild transient dysarthria and hand kinetic tremor
Richard S. et al. 2018 [37]	Medial Thalamus	13	16–33	5	12	6–58 mo	Retrospective review	1.YGTSS 50% improvement	1.OCD (Y-BOCS) (*n* = 12) 63% patients > 50% improvement 2.Clinical Global Impression scale much or very much improve	1.Device-related: wound erosion and infection
Daniel.et al. 2016 [38]	Ventral anterior and ventrolateral motor parts of the thalamus	8	19–56	0	2	12 mo	Retrospective open-label trail	1.YGTSS 58% improvement motor scores 51% improvement vocal scores 53% improvement impairment 60% improvement 2.MRVRS 58% improvement	1.OCD (Y-BOCS) no significant improvement 2.Depression (BDI) no significant improvement 3.Anxiety (STAI) trait anxiety significant improve state anxiety no significant improve 4.DAPP-BQ: no effect on personality dimensions of emotional dysregulation, dissocial behavior, inhibition and compulsivity 5.GAF: a significant effect on the patients’ overall level of functioning 6.Modular System for Quality of Life: significant effect on the patients’ general satisfaction with life, psychosocial quality of life, and their affective quality of life	1.Surgery-related: NR 2.Postsurgical adverse effects infection of the IPG pouch 3.Stimulation-related: weight gain, tic severity increase, consecutive deterioration of mood, disturbance of sleep, dysarthria, feelings of heaviness, heat, headache, a humming feeling during head rotation, feelings of a sudden twitch or twinge, agitation and loss of strength in one leg, numbness and tremor of the tongue, lower jaw, and cramps of the hands; intensity increase result dysarthria, disturbance of eye motility and fine motor skills; intensity decrease result disturbance of eye motility and tremor of the lower jaw
Anouk Y.J.M et al.2016 [39]	Cm-Spv-Voi; GPi	7	35–48	NR	NR	12–78 mo	Case series	1.YGTSS 27.5–88.9% improvement	NR	1.Surgery-related: vertical gaze paralysis (bleeding) 2.Postsurgerial adverse effects infection of IPG, binge eating, lethargy, dysarthria, gait disturbances and apathy 3.Stimulation-related sleeping disorders, gaze disturbances; intensity increase result reduced level of energy, minor visual disturbances, and alteration of sexual function
Zinovia et al. 2015 [40]	GPi	15	24–55	0	1	8–36 mo	Randomized, double-blind, crossover trial	1.YGTSS blinded phase (on-stimulation vs stimulation-off): 15.3% improvement baseline vs open-label stimulation phase: 40.1% improvement	Baseline vs open-label stimulation phase 1.OCD (Y-BOCS) no significant change 2.Depression (BDI) significantly improve 3.Axiety (STAI) no significant change 4.GTS-QOL significantly improve	1.Surgery-related: infection of the hardware 2.Stimulation-related deterioration of tics and hypomanic behavior
Elisabeth et al. 2012 [41]	amGPi	11	18–50	0	2	4–30 mo	Retrospective review	1.YGTSS 49.6% improvement at 3 mo motor score 48% improvement at final follow-up vocal score 56.5% improvement at final follow-up	1.OCD (Y-BOCS) (*n* = 9) 59% improvement at 3 months 2.Depression (HAM-D) (*n* = 6) 74% improvement at 3 months 3.GTS-QOL change from 39.09 to 79.09 at final follow-up 4.GAF change from 47.27 to 74.55 at final follow-up	1.Surgery-related: NR 2.Device-related lead breakage or damage, lead infection 3.Stimulation-related: anxiety with panic attacks
Perminder S. et al. 2014 [41]	amGPi	17	17–51	2	6	8–46 mo	Retrospective review	1.YGTSS 54.3% improvement motor score 47.8% improvement vocal score 51.5% improvement	1.OCD (Y-BOCS) (*n* = 11) change from 13.88 to 5.29 2.Depression (HDRS)(*n* = 8) change from 15.35 to 8.00 3.GTS-QOL change from 40.88 to 66.47 4.GAF change from 50.0 to 72.12	1.Surgery-related: NR 2.Device-related: lead breakage or damage, lead infection 3.Stimulation-related: transient anxiety, agitation, dizziness, poor balance and worsening of pre-existing stuttering, intermittent speech arrest
Johnson et al. 2019 [42]	CM thalamus; anterior GPi; posterior GPi; NA/ALIC	123	14–61	NR	NR	1–120 mo	Multisite study	1.YGTSS 46.7% improvement 2.Median time to clinical response (≥40% reduction in YGTSS) CM thalamus: 12 mo GPi:18 mo all patients: 13 mo	1.OCD (Y-BOCS) 21.1% improvement 2.Median time to clinical response (≥40% reduction in YGTSS) TS with OCD: 24 mo TS without OCD: 11 mo	NR
A.Y.J.M Smeets et al. 2016 [43]	anterior GPi	5	35–57	0	0	12–38 mo	Retrospective review	1.YGTSS motor score s64.8% improvement vocal scores 78.2% improvement 2.MRVRS motor scores 79.7% improvement vocal scores 81.0% improvement	1.Anxiety (BAI) no significant change 2.Depression (BDI) no significant change 3.ADHD (CAARS) no significant change 4.OCB (Y-BOCS) no significant change	1.Postsurgery adverse effects: infection of IPG and neck pain 2.Stimulation-related:apathy, weight loss and agitation
Hauseux et al. 2017 [44]	posteroventral GPi; posteroventral GPi + NA	3	12–18	2	3	40–69 mo	Case series	1.YGTSS motor tics improved(n = 2), vocal tics remained(n = 1) or exacerbation(*n* = 1), and no significant effects on tics(*n* = 1)	1.OCD no improvement(n = 1), exacerbation(n = 1) or recurrence(n = 1) 2.GTS-QOLmoderate(*n* = 2) or low score(n = 1)	1.Stimulation-related: exacerbation of phonic tics and OCD, dysarthria, recurrence of severe depressive symptoms and self-injurious behaviors
Fabian et al. 2013 [45]	GPe	1	47	0	0	6 mo	Case report	1.YGTSS 70.5% improvement	1.Anxiety (HARS) 75%improvement 2.Depression (HDRS) 82.3% improvement 3.GAF score 36.4% improvement 4.MMSE scores 17.4% improvement	1.Stimulation-related:battery depletion and loss of stimulation
Jens et al. 2008 [46]	NAc/ALIC	1	26	0	0	10 mo	Case report	1.YGTSS 20% improvement at 4 weeks 50% improvement at 10 months	NR	1.Stimulation-related: manic-like state (euphoric mood and elation, partially inappropriate behavior, overly familiar interaction patterns, restlessness, psychomotor agitation, and mild logorrhea)
Perminder Singh et al. 2012 [47]	NAc	1	32	0	0	8 mo	Case report	1.YGTSS 57% improvement at 1 month 79% improvement at 7 months	1.OCD (Y-BOCS) 90% improvement at 1 month 68% improvement at 7 months	NR
Irene et al. 2009 [48]	NAc/ALIC	1	38	0	0	36 mo	Case report	1.YGTSS 46% improvement at 3 months 44% improvement at 36 months 2.MRVRS 60% improvement at 3 months 58% improvement at 36 months	1.OCD (Y-BOCS) 53% improvement at 3 months 56% improvement at 36 months	NR
Irene et al. 2010 [49]	NAc/ALIC	1	42	0	0	36 mo	Case report	1.YGTSS 44% improvement 2.MRVRS 58% improvement	1.OCD (Y-BOCS) 56% improvement	NR
Adam et al. 2010 [50]	NAc/ALIC	1	22	0	0	30 mo	Case report	1.YGTSS 15% worsen	OCD (Y-BOCS) no significant improvement	NR
Clemens et al. 2017 [51]	Field H1 of Forel	2	19–31	0	1	6–18 mo	Case series	1.YGTSS patient 1: 91.1% improvement tic severity 82.1% improvement impairment 100% improvement patient 2: 62.7% improvement tic severity 36.4% improvement impairment 83.3% improvement	1.Depression (BDI) patient 1100% improvement patient 2 89.7% improvement 2. Anxiety (STAI) State anxiety (STAI-X1) patient 1 63.9% improvement patient 2 38.3% improvement Trait anxiety (STAI-X2) patient 1 63.8% improvement patient 2 38.8% improvement 3.OCD (Y-BOCS) patient 1 93.8% improvement patient 2 slightly reduction 4.MSQoL patient 1 53.1% improvement patient 2 43.1% improvement 5.GAF change from serious to minimal impairment	1.Stimulation-related: patient 2: worsening of tics
Irene et al. 2009 [52]	STN	1	38	0	0	12 mo	Case report	1.Tics frequency 89% improvement at 6 mo 97% improvement at 1 year	NR	NR
Domenico et al. 2016 [53]	CM-Pf-Voi, NA-ALIC, amGPi, pvGPi	37	17–57	1	8	8–32 mo	Retrospective review	1.YGTSS all patients: 42.0 reduction CM-Pf-Voi(*n* = 27): 47.5 reduction	1.Y-BOCS> 16 and BDI < 19(n = 7) OCD (Y-BOCS): improvement(n = 6) or slight worsening(n = 1) 2.Y-BOCS< 16 and BDI > 19(n = 6) Depression (BDI): improvement(n = 5) or exacerbation(n = 1) 3.Y-BOCS> 16 and BDI > 19:(*n* = 17) OCD (Y-BOCS): improvement(*n* = 16) or worsening(n = 1) Depression (BDI): improvement(*n* = 17)	1.Device-related: inflammatory reaction to the DBS system, infection, wall hematoma in the IPG pouch, skin erosion
B.Kakusa et al. 2019 [54]	CM-Pf complex+ VC/VS	1	20	0	1	12 mo	Case report	1.YGTSS 84% improvement	1.OCD (Y-BOCS) 70% improvement 2.Depression (HDRS-D17) 95% improvement	1.Stimulation-related: sensory disturbances and dizziness
Raphaëlle et al. 2018 [55]	1st: pvl GPi 2nd: ventral anterior and ventrolateral motor regions of the thalamus 3rd: radiosurgery: ventral portions of the ALIC	1	47	0	0	6 yrs	Case report	1.YGTSS 1st: no change 2nd: 69% improvement	1.OCD(Y-BOCS) 1st: 40% improvement 2nd: 40% improvement 3rd: 70% improvement 2.QIDS-SR 16 2nd to 3rd: 21 to 9 3.GAF 2nd to 3rd: 22 to 87	1.Stimulation-related: weight gain
Zhang et al. 2019 [56]	GPi and anterior capsulotomy	10	19–43	0	6	24–96 mo	Retrospective review	1.YGTSS 77% improvement motor scores 75% improvement vocal scores 78% improvement 2.CGI-SI score 71% improvement	1.OCD (Y-BOCS) 87% improvement 2.Depression (HAMD-24) 93% improvement 3.Anxiety (HAMA) 94% improvement 4.ADHD (ADHD-RS-IV) 28% improvement 5.GAF score 134% improvement 6.GTS-QQL 92% improvement	1.Stimulation-related: fatigue, laziness, confusion, disorientation; an episode of inarticulate speech and transient epileptic seizure 2.Device-related: extension wire–related infection
Zhang et al. 2019 [57]	pv GPi and capsulotomy	1	20	0	1	3 mo	Case report	1.YGTSS 53% improvement tics 45% improvement impairment 60% improvement	1.OCD (Y-BOCS) 42% improvement 2.Anxiety (BAI) 20% improvement 3.Depression (BDI-II) 62.5% improvement	NR
Anouk Y. J. M et al. 2018 [58]	anterior GPi	2	19	0	2	2 yrs	Case series	1.YGTSS patient 1: 53% improvement at 2 years motor scores 43% improvement vocal scores 60% improvement patient 2: 69% improvement at 1 year motor scores 65% improvement vocal scores 78% improvement	NR	1.Stimulation-related: patient 1: hyperkinesia, dyskinesia in the legs and a dejected mood patient 2: increased agitation, an increase in tic frequency and severity
Rene et al. 2018 [59]	CM-Pf	1	27	0	0	12 mo	Case report	1.YGTSS scheduled stimulation 33% improvement responsive stimulation 48% improvement 2.MRTRS scheduled stimulation 53% improvement responsive stimulation 64% improvement	NR	NR

**Abbreviations:** *ADHD* attention deficit hyperactivity disorder; *amGPi*, anteromedial or limbic, *GPi ALIC* anterior limb of internal capsule, *BAI* Beck Anxiety Inventory, *BDI* Beck Depression Inventory, *CAARS* Conner’s Adult ADHD Rating Scale, *CM-Pf-Voi* centromedian-parafascicular-ventro-oral internus complex, *CGI-SI* Clinical Global Impression–Severity of Illness scale score, *DAPP-BQ* Dimensional Assessment of Personality Pathology–Basic Questionnaire, *FIQ* Full scale Intelligence Quotient score, *GAF* Global Assessment of Functioning Scale, *GTS-QOL* Gilles de la Tourette Syndrome-Quality of Life, *HARS* Hamilton Anxiety Rating Scale, *HDRS* Hamilton Depression Scale, *MMSE* Mini-Mental State Examination, *mo* month, *MRVRS* Modified Rush Video-Based Rating Scale, *NAc* nucleus accumbens, *NR* not reported, *OCD* Obsessive-Compulsive Disorder, *plGP/pvGPi/pvlGPi*, posterolateral/posteroventral/posteroventrolateral, *GPi, QIDS-SR16* Quick Inventory Depression Scale – Self Report 16, *SCL90* Symptoms Checklist List 90, *STAI* State-Trait Anxiety Inventory, *STN* subthalamus nucleus, *VC/VS* ventral capsule/ventral striatum, *Y-BOCS* Yale-Brown Obsessive Compulsive Scale, *YGTSS* Yale Global Tic Severity Scale, *yr* year

### Single target

#### Thalamus

To date, the majority of TS DBS treatment studies has focused on the thalamus due to its strategic location between motor areas of the cerebral cortex and motor-related subcortical structures, particularly the basal ganglia and cerebellum [33, 34]. A retrospective study of several patients with refractory TS and psychiatric comorbidities reported that DBS of the thalamic centromedian-parafascicular (CM-Pf) complex was associated with a 46% improvement in motor tics and 52% improvement in phonic tics, as measured by the Yale Global Tic Severity Scale (YGTSS) at follow-up (mean duration: 26 months) [34]. Moreover, DBS of this thalamic region markedly improved the patients’ social, occupational, and educational functioning. Furthermore, two case studies reported that DBS of the thalamic CM-Pf improved comorbid OCD, anxiety, and depression, as assessed by the Yale-Brown Obsessive Compulsive Scale (Y-BOCS), Beck Anxiety Inventory (BAI), Symptoms Checklist List 90 (SCL90), and Beck Depression Inventory (BDI) [35, 36]. Several other case reports and case series have reported that DBS of the CM-Pf region can alleviate motor tic severity as well as comorbid psychiatric symptoms in patients with TS [37, 38].

In addition, one study reported that DBS of the medial thalamic region produced a mean 50% improvement in overall tic severity (YGTSS total score) at 6-month follow-up [37]. Interestingly, the active lead location was in the region of the posterior ventralis oralis internus/CM-Pf complex [37], suggesting that the CM-Pf complex may have partially mediated the beneficial effects of the medial thalamus DBS on TS symptoms. This effect on TS symptom severity could stem from the modulation of excitatory fibers of the CM-Pf projecting to the striatum and subthalamic nucleus, although this hypothesis remains speculative [60]. In contrast to its effect on tic severity, DBS of the medial thalamus did not produce an overall, group mean improvement in the patients’ Y-BOCS scores [37]. However at the individual level, about 63% of the patients with TS achieved a greater than 50% reduction in their Y-BOCS scores and one patient experienced an increase in OCD symptoms [37]..

In a prospective open-label trial, DBS of the ventral anterior and ventrolateral motor parts of the thalamus was similarly effective in reducing tic severity in 8 patients with TS and psychiatric comorbidities [38]. Additionally, DBS improved the patients’ anxiety, adaptive functioning, and quality of life,owever, no significant effects were observed on comorbid symptoms of OCD (Y-BOCS), anxiety (State-Trait Anxiety Inventory, STAI), and depression (BDI) [38]. Thus, these studies suggest that DBS of each thalamic region can reduce tic severity and to some extent, improve comorbid anxiety and depression [61]. Only DBS of the CM-Pf has been reported to alleviate comorbid OCD symptoms in some cases of TS.

In general, DBS of the thalamus has been well tolerated, but patient risk and adverse side effects remain an issue of concern. Reported side effects include the transient blurring of vision, dysarthria, recurrent tension headache, and a single seizure-like episode (after DBS of the CM-Pf [34, 36]). Disturbances of eye motility have also been documented, as well as impaired fine motor skills, particularly following DBS of the ventral anterior and ventrolateral motor thalamic regions [38]. Motor side-effects of thalamic stimulation is likely larger given that it occurs as the simulation increases. Emotional disturbances, erectile dysfunction, paresthesia, weight gain, and apathy may also be observed in some patients after thalamic DBS [27]. Of note, the development of the latter side effect is somewhat surprising as apathy has traditionally been linked to lesions of basal ganglia structures altering the cortico-striatal-pallidal-thalamic-cortical pathways [62]. In some patients, the side effects associated with thalamic DBS can outweigh its therapeutic benefit over the long-term course of treatment, necessitating the exploration and modulation of a target other than the thalamus for the patients with TS [39].

#### Globus Pallidus

The globus pallidus (GP) is a promising DBS target for managing severe and refractory TS [63]. As alluded to earlier, the GP is an element of the basal ganglia-thalamocortical circuit that is believed to play a crucial role in the control of motor function. The GP, consisting of the internal segment (GPi) and the external segment (GPe), participates in both the direct and indirect motor pathways. Some experts have hypothesized that the GP modulates the excitability of the thalamus and influences the input from thalamus to cortex [64]. A recent resting-state functional magnetic resonance imaging (fMRI) study indicates that the GP could be involved in TS pathophysiology [63],owever, the putative role of the GP in TS remains to be clearly defined.

A randomized, double-blind, crossover clinical trial assessed the utility of bilateral GPi DBS in alleviating TS motor symptoms [40]. In this study, 14 patients were randomly allocated to receive either stimulation on-first or stimulation off-first for 3 months, followed by a switch to the opposite condition for an additional 3-month period. Thirteen patients completed assessments during both blinded treatment conditions. The results revealed that tic severity of these patients, as determined by the mean YGTSS total score, was reduced by an approximate 15% (95% CI: 5–25%) during the on-stimulation period compared to the off-stimulation period. Moreover, bilateral anterior GPi DBS reduced the severity of comorbid depression (BDI) compared to baseline prior to surgery. The stimulation had no significant effects on comorbid OCD symptoms (Y-BOCS) and anxiety (BAI) during an open-label period [40].

DBS of the limbic or anteromedial GPi (amGPi) has been successfully applied to TS treatment. In one study, 15 patients with severe and refractory TS were treated with amGPi DBS and clinically assessed before surgery and between 17 and 82 months after surgery [32]. The results showed that the patients’ tic severity was significantly reduced at follow-up (mean reduction of YGTSS total score: 38%; phonic score: 38%; motor score: 33%) [32]. At the group level, amGPi DBS had no significant effect on comorbid OCD (Y-BOCS), depression (BDI), and anxiety (BAI). However, the authors identified a subgroup of patients with severe baseline OCD symptoms as defined by Y-BOCS who had a 39% improvement after amGPi DBS [32].. Although this study found no overall effect on depression, other studies have reported an improvement in comorbid depression following amGPi [41, 42, 65]. In another study, DBS was targeted on the anterior GPi, which produced a significant tic improvement but failed to alleviate the patients’ comorbid anxiety and depression [43].

A multicenter study of TS patients with medial GPi DBS reported improvements in tic severity, comorbid OCD, anxiety, depression andquality of life [42]. The median time to achieve a clinical response (≥ 40% reduction in YGTSS total score) was 13 months. In this study, the clinical outcomes of TS patients treated with GPi were compared with the outcomes of patient who had been treated with DBS of other targets, including the CM thalamus. No significant differences in the strength or timing of the clinical response were observed across the different DBS targets, although the response to GPi stimulation wasslower than thalamic CM stimulation (18 months, 95% CI: 12–24 vs 11 months, 95% CI: 6–15). Finally, a retrospective study reported that posteroventral GPi DBS improved motor tics in 3 teenagers with refractory TS [44]. Posteroventral GPi DBS also stabilized comorbid OCD symptoms present in one patient. These findings suggest that posteroventral GPi DBS could serve as a safe and effective intervention for managing both tic and OCD symptoms in select adolescent patients who suffer from TS.

GPi DBS has been associated with various adverse events and side effects. For example, 3 patients (out of a total of 13 patients) experienced significant adverse events (2 patients developed DBS hardware-related infections and 1 patient a DBS-induced hypomania) following amGPi DBS [40]. All adverse side effects were managed or resolved over the treatment course. Also, amGPi DBS has been associated with weight gain, dizziness, feelings of nausea, freezing-of-gait episodes, impaired speech articulation, and akathisia [32]. Similarly, posteroventral GPi DBS has been associated with dysarthria [44], dystonia, and dyskinesias [4].

The prior studies reviewed have focused on the GPi but have not explored the GPe as a potentially effective DBS target for TS treatment. A recent study examined tonic and phasic neuronal activities in the anterior GPe and GPi in 8 awake patients with TS while DBS electrodes were implanted [66]. The results showed that the expression of tics was accompanied by tonic and phasic changes of neuronal activity throughout the GP. A large fraction of both GPe and GPi neurons changed their baseline firing rate around the time of the tics, indicating that both GP segments could have a role to play in TS pathophysiology. Indeed, a case report described a 47-year-old patient with refractory TS who showed marked improvements in tics and mental health status following bilateral GPe DBS [45]. Moreover, when the stimulation was unexpectedly interrupted due to battery depletion, some of the patient’s TS symptoms reemerged. These findings suggest that the GPe can also be considered as a potentially effective DBS target for managing severe and refractory TS.

#### Nucleus accumbens and anterior limb of the internal capsule

A few case studies have assessed the utility of DBS of the nucleus accumbens (NAc) and the anterior limb of the internal capsule (ALIC) in TS treatment. One report of a 26-year-old patient with TS had a 50% reduction in tic severity after bilateral NAc DBS [46]. Other case reports confirmed the beneficial effects of DBS of the NAc, as well as of the ALIC, on the severity of tics [47, 48]. In one of these case studies, the patient had a 57% reduction in tic severity (assessed by the YGTSS) and a 90% reduction in OCD symptom severity (Y-BOCS) at 1-month follow-up [47]. Similarly, another case report of a 38-year-old TS patient reported a 53% reduction in OCD symptoms at 3-month follow-up, which was sustained until 36-month follow-up [48]. In the latter study, however, the patient continued experiencing recurrent depressive episodes [49]. This observation highlights a caveat to the treatment, namely that DBS of the NAc/ALIC region may induce affective side effects, including both depression and hypomania [46].

In conclusion, all the brain targets reviewed so far have shown some effectiveness in managing severe and refractory TS. A recent meta-analysis (57 studies, including a total of 156 patients) showed that DBS treatment was associated with an overall 53% improvement in tic severity scores on the YGTSS, with no significant differences between the targets examined (thalamus, posteroventrolateral part and the anteromedial part of the GPi, NAc, and ALIC) [27]. Data from the TAA Registry are in line with these results [4].

#### Other targets

Some studies reported clinical improvements in patients with TS when the DBS was targeted on the junction of multiple adjacent thalamic nuclei [27, 38]. An alternative target involves the Forel’s field H1, through which the projections from GPi to thalamus pass. This area was found to be an effective and well-tolerated alternative target in two cases of refractory TS [51]. According to the authors, stimulation of the H1 field could normalize a decreased output of the GP through retrograde stimulation of the GPi. The authors further speculate that DBS of Forel’s field H1 could help to restore the balance between the direct, indirect, and hyper-direct motor pathways, ultimately limiting excessive activity of the thalamo-cortical network in TS. Moreover, DBS of Forel’s H1 field has been found to relieve comorbid depression and anxiety in two cases of refractory TS [51]. In one of the two cases, the stimulation of this target also improved the patient’s comorbid OCD symptoms. Targeting Forel’s H1 field has the advantage over direct thalamic stimulation because DBS of the H1 field can be performed at low stimulation intensities, reducing both stimulation-related adverse events and battery depletion. However, the precise anatomical localization of this region is difficult to identify using current imaging or other neurophysiologal techniques, limiting its potential clinical use at the present time.

Finally, the subthalamic nucleus (STN) is the most common target for DBS treatment for Parkinson disease (PD), but some evidence exists that this target may also be effective for managing TS symptoms. For example, it has been reported that a 38-year-old patient with PD who also suffered from TS showed a 89% improvement in tic frequency after 6 months and a 97% improvement after 12 months of bilateral STN-DBS treatment [52]. This report indicates that STN DBS may modulate dysfunction of both limbic and sensorimotor areas and that this stimulation may provide a quicker relief of tics than seen following medial thalamus or GPi stimulation. In another study, 4 patients with TS received DBS of both the bilateral GPi and bilateral STN. The researchers also obtained recordings of local field potentials and the electromyogram from the patients between 3 and 5 days after DBS implantation [56]. The results were taken to indicate that STN and GPi stimulation can improve acute TS symptoms by modulating neuronal oscillations in the basal ganglia. However, the GPi DBS showed a better clinical effect on OCD than STN DBS. Nonetheless, the available database is extremely small and further studies are required to assess whether or not the STN is an effective DBS target for TS treatment.

### Multiple targets

It has become increasingly clear that DBS of a single target is insufficient to manage the clinical symptoms of all patients, given the heterogeneity and complexity of the TS syndrome itself and the presence of large inter-individual differences in clinical response to DBS treatment. For certain symptoms, the use of multiple targets could have a more effective or widespread effect compared to the use of a single target. For example, DBS of multiple targets could aid in managing severe psychiatric comorbidities in some select patients with TS. A recent case report illustrates the feasibility and effectiveness of such a strategy [54]. In this study, DBS targeting simultaneously the CM-Pf complex and ventral capsule/ventral striatum (VC/VS) was found to produce widespread clinical benefits in a 20s male patient with TS and comorbid major depressive disorder, OCD, and opioid use disorder. The patient’s YGTSS, YBOCS, and Hamilton Depression Scale (HAMD) scores were improved by 84, 70, and 95%, respectively, after one year of bilateral, dual-target DBS. Also, the patient’s dependence for opiate medications was improved and he had self-tapered off the medication [54].

Multiple DBS targets also play a role in “rescue” DBS treatment, where the patient receives a second lead placement in a different target following a suboptimal clinical response to the initial surgery [53]. Although the use of multiple targets may have clinical value, this strategy carries an increased surgical risk, risk of adverse side effects, and complications relative to the use of a single target. Therefore, a clear understanding of the benefits and risks associated with the use of multiple targets, along with adequate patient selection is required when adopting this therapeutic strategy.

### DBS combined with radiosurgery to address psychiatric comorbidity

To date, only a few studies have explored the use of DBS combined with stereotactic radiosurgery for managing refractory TS and psychiatric comorbidities. A recent case study [55] illustrates the potential utility of this treatment strategy. In this study, a 47-year-old female patient with refractory TS and comorbid OCD had a poor clinical response (YGTSS = 39/50, Y-BOCS = 28/40) to her first surgical treatment involving posteroventrolateral GPi DBS. One year after the first surgery, a second DBS device was implanted in the contralateral ventral anterior and ventrolateral motor regions of the thalamus, which produced a significant improvement in motor and vocal tics (YGTSS = 10/50) but did not change the severity of her OCD symptoms (Y-BOCS = 28/40). Two years after the second DBS surgery, the patient received gamma knife surgery targeting the ventral portions of the ALIC. Following this intervention, the severity of her comorbid OCD symptoms was greatly reduced at 9-month follow-up. The patient was in clinical remission at 12-month follow-up (Y-BOCS = 6/40). The remission of her OCD was accompanied by improvements in depressive symptoms [55]. This case report implies that DBS combined with radiosurgery could alleviate severe psychiatric comorbidities in select cases of TS.

This notion seems to be supported by a retrospective study of 10 patients with refractory TS and psychiatric comorbidities [57]. In this study, patients had been treated with GPi DBS combined with bilateral anterior capsulotomy. The results showed significant improvements in patients’ motor and verbal tics (YGTSS), as well as in the severity of their comorbid psychiatric disorders, mainly consisting of OCD and affective disorders. Moreover, the patients’ social functioning and quality of life were substantially improved after the combined neurosurgical treatment [57]. In addition to these results, GPi DBS combined with capsulotomy may also offer an effective, rapid, and tolerable intervention for rare cases of “malignant” TS [67].

## Surgical candidates

Appropriate patient selection for DBS surgery requires a careful multidisciplinary approach. Both treatment refractoriness and symptom severity are important eligibility criteria for DBS. For example, some patients with treatment-resistant profound tics, self-injurious behavior or even life-threatening symptom [13, 44, 68] TS is a severely disabling clinical condition that warrants consideration of neurosurgical intervention. According to the recommendations of the TAA published in 2006 [17], only patients who are older than 25 years should be eligible for DBS trials, although the risk of surgical complications and adverse events does not appear to be greater among reported cases of TS under 25 years of age who had documented DBS [17]. The TAA’s updated recommendation in 2015 [58], no longer specifies an age limit for DBS trials. A multidisciplinary team that careful considers the medical and ethical issues involved in DBS treatment should guide patient selection while ensuring patient rights, safety, and care.

The American Academy of Neurology (AAN) has recently published recommendations for the optimal management of TS [58, 68]. This includes the use of a multidisciplinary screening team pre- and post-operatively, offering cognitive behavioral therapy to patients, screening for psychogenic/functional tics, and mental health assessments conducted by a psychiatrist pre- and post-operatively to confirm the DSM-V diagnosis and assess psychiatric comorbidities.

### Effects of DBS in childhood

A retrospective case series reported the clinical outcomes of 13 patients treated with medial thalamic DBS for refractory TS [37]. The average age was 20 years (12 patients were younger than 25 years and 1 patient was 33 years old). After DBS, the patients continued to have tics but the overall severity of tics (YGTSS total score) was reduced by 50% at the last (6–58 months) follow-up. Adverse side effects and complications reported included skin erosion, skin infection at the connector site, headache, and changes in mental state secondary to obstructive hydrocephalus. A prospective case series examined the outcomes of 3 patients with TS (19–21 years old) treated with DBS of the bilateral CM-Pf-ventral oral nuclei [35]. One year after DBS, the patients showed significant reductions in tic severity and social impairment. The patients’ levels of intelligence did not change after treatment. Another study evaluated the long-term clinical outcomes of 3 adolescents, including the then youngest patient worldwide (12 years old at the time of surgery), who underwent posteroventral GPi DBS for managing refractory TS [44]. After DBS, the pediatric patients exhibited a substantial improvement in motor tics, although their phonic tics and psychiatric comorbidities were not affected. In another study, the tics of 2 patients (both 19 years old) were improved, at least to some extent, after anterior GPi DBS [69].

Recently, a meta-analytis review of individual patient data from DBS studies with children and adolescents with refractory TS (*N* = 58; aged 12–21 years) has been published [70]. The studies reviewed targeted the thalamus or GPi regions. The results showed that across patients, studies, and targets, DBS treatment was associated with a reduction in tic severity (YGTSS) by about 58% (SD = 25; *p < 0.001*). Moreover, DBS treatment was associated with a reduction in comorbid OCD symptoms (YBOCS) by 31% (SD = 45; *p < 0.001*) and anxiety (STAI) by 40% (SD = 20; *p < 0.001*) [70]. Although both targets were associated with significant tic improvements, greater improvements in tic severity were observed in thalamic stimulation compared to pallidal stimulation (YGTSS score improvement: thalamus: 69%; GPi: 53%; *p = 0.0387*), especially in patients with less severe TS symptoms at baseline. Additionally, the presence of comorbid depression was associated with a less favorable response to DBS treatment. Side effects were noted in about 28% of the patients however most were considered mild. The main results from this meta-analysis are congruent with the data from the TAA Registry (including data from pediatric patients aged 13 years and older) [4].

In summary, DBS is a treatment option for adolescent patients who suffer from severe and intractable TS and have undergone careful assessment and selection by a multidisciplinary team [17, 58, 69, 70]. Early DBS intervention in younger patients is still controversial, given the possibility of resolution of symptoms later in life without DBS. Some experts argue that even though the possibility of symptom resolution at a later age exists, an earlier DBS intervention in select pediatric patients may improve their social adjustment and clinical outcomes [71]. More studies are needed to shed light on this important issue.

### Ethics of DBS in childhood

Several ethical issues need to be considered in DBS treatment of pediatric patients with TS. As indicated in the prior section, one important ethical question is whether or not DBS should be considered in a teenager with TS, given that tics decrease in 40% of TS patients and disappear completely in another 40% of patients during adolescence and young adulthood [2]. Based on new insights and revised guidelines [17], patient’s age is no longer a strict eligibility criterium for DBS treatment. Instead, the eligibility for DBS treatment should be based on a careful assessment of the benefits and risks of the neurosurgical intervention for a given patient.

For some pediatric patients, the benefits of DBS can outweigh the risks associated with the intervention. As discussed in the prior section, DBS can offer substantial clinical benefits to patients who suffer from otherwise intractable TS with severe psychiatric comorbidities, self-injurious behavior or even life-threatening symptoms [13, 44, 67, 68]. Another argument in favor of early DBS intervention is that severe TS in adolescence is associated with a high risk of bodily harm, disrupted cognitive and emotional development, low self-esteem, and poor quality of life [69]. This situation can jeopardize educational and job opportunities, social interactions, and relationships with peers. Thus delaying surgery in these young patients could result in permanent harm to their cognitive, emotional, and social development, even if the TS symptoms eventually subside with age. On the other hand, DBS is an invasive treatment with potential surgical complications and many adverse side effects.

Other factors are also relevant for determining whether an adolescent patient may be a reasonable candidate for DBS. These include psychosocial factors, such as the presence or absence of a stable and supportive social environment, as well as psychological factors, such as the patient’s individual resilience and coping strategies. Voluntary written informed consent must be obtained from the pediatric patient and/or the legal guardian before DBS treatment [69]. DBS is a potentially powerful treatment for managing the clinical symptoms of TS and its psychiatric comorbidities in select patients who do not clinically respond to conventional treatments. Finally, studies have provided evidence that DBS can improve the motor and vocal tics in TS. An important goal of future DBS treatment studies is to improve the patient’s clinical symptoms along with improving his or her functional impairments and quality of life.

There is substantial inter-individual variability in the clinical response to DBSA current clinical challenge lies in finding a marker to predict the patient’s clinical response to DBS. To date no genetic, biological, behavioral or other type of marker has been identified that can accurately predict the clinical response to DBS for individual patients. Longitudinal prospective studies, involving large cohorts, standardized surgical procedures, and multidimensional assessment protocols will be needed to identify potential prognostic markers [44].. Finally, DBS treatment for TS seems to be associated with a higher infection risk [72]. Additional research is also needed to determine whether the risk of infection or its complications differ between younger and older patients.

## Optimization of DBS treatment

Chronic high-frequency stimulation has been associated with long-term improvement in motor and vocal tics across multiple targets. It is unknown whether some patients with TS develop tolerance to continuous stimulation or experience disease progression over the course of the long-term treatment. The TAA Registry and other studies have documented the adjustments made to the stimulation parameters (e.g., increasing pulse voltage) to maintain control of tics following DBS surgery [4, 32]. Such adjustments are made in an effort to maintain symptomatic control but they could increase the total energy delivered to the patient, thereby draining the battery more quickly, leading to more frequent battery replacements and increasing the burden on the patient involved. In the near future, this problem may be resolved by rechargeable technologies. The development and use of rechargeable technologies could also resolve the increasing difficulties in obtaining insurance and healthcare authorizations to pay for battery replacements [73].

### Adaptive deep brain stimulation

At present, most DBS systems function in an ‘open-loop’ mode, that is, the stimulation parameters are preset in advance and cannot be changed or updated according to the clinical symptoms of the patient or to underlying pathophysiological changes in the brain. The classical open-loop system, however, represents a static approach to therapy within an inherently dynamic system [74]. In contrast, responsive or adaptive DBS (aDBS) is designed to function as a ‘closed-loop’ stimulation device, which can be personalized according to the frequency and duration of a physiologic event or behavioral manifestation [59, 75–78]. The closed-loop system in which stimulation is dependent on functional neural feedback were initially designed to improve the treatment of epilepsy [79, 80]. Recent studies suggest that aDBS is a more effective approach than conventional DBS to treat epilepsy and other neurological disorders, including PD [81, 82] and essential tremor [83, 84].

The primary goal of aDBS is to widen the therapeutic window. As opposed to closed-loop systems, aDBS can be used to deliver stimulation according to the current state of pathological activity, as indexed by real-time changes in the patient’s brain electric signals. This method may avoid the unwanted situation that stimulation is given to patients when they are in a healthy, tic-free state [78]. For DBS, the measurement of local field potential (LFP) activity has been favored over microelectrode recordings of single neurons given that LFPs can be readily measured from the implanted DBS leads [85, 86]. In one study, certain LFPs in the thalamus closely linked to the generation of tics were identified in 3 patients with severe and intractable TS while undergoing thalamic DBS [87]. Correspondingly, in line with the putative role of the thalamus in TS pathophysiology, it may be hypothesized that the monitoring of thalamocortical network activity could be useful in aDBS to detect the presence of tics and associated pathological activity in patients with TS [88].

Indeed, a case report has recently provided the first evidence for the utility and feasibility of aDBS in TS treatment [75]. In this study, a 27-year-old patient with intractable TS was treated first with conventional, continuous DBS of the CM-Pf. After four years of stimulation, the battery was depleted and surgically replaced. On this occasion, the patient’s implant involved aDBS so that stimulation was given only when tic-related, pathological activity occurred in the CM-Pf. One year later, the patient’s scores on the YGTSS and Modified Rush Tic Rating Scale (MRTRS) were improved by 48 and 64%, respectively, when compared to the scores observed before aDBS implantation surgery. These data not only support the clinical utility of aDBS but also indicate that this type of stimulation could be more effective than conventional DBS for refractory TS.

A secondary goal of aDBS has been to reduce power drain on the battery/neurostimulator (IPG). Rechargeable IPG systems are unsuitable for a significant proportion of patients [89]. Moreover, those patients who use them would benefit if recharging occurred less frequently. In this context, it is interesting to note that efforts are being devoted to reducing rechargeable battery size sufficiently to make skull-mounted IPGs possible [90]. In the case study discussed earlier, it was observed that the use of aDBS resulted in a 63% improvement in the neurostimulator’s projected mean battery life when compared with scheduled stimulation [75]. In addition, there was a 145% improvement when compared with duty-cycle-only therapy. The cumulative stimulation dosage was also calculated. The calculated reductions in the duty cycle and the scheduled duty cycle schemes were 40 and 80%, respectively. The daily dosage, which refers to the cumulative on time of the devices, for the open-loop, duty-cycle, scheduled duty-cycle, and responsive paradigms corresponded to 24, 2.82, 0.94, and 0.56 h, respectively, signifying that the estimated battery life could be extended to 2.5 years for responsive stimulation [75]. Thus, these data suggest that the use of aDBS treatment for TS could also yield long-term economic and practical benefits.

### Functional connectivity profiles

No marker has so far been identified that accurately predicts the clinical response of patients with TS to DBS treatment. Recently, resting-state fMRI studies have reported some intriguing findings that could lead to the development of a prognostic marker. These studies have focused on the structure and function of the so-called ‘default mode network’ (DMN), which refers to a widely distributed brain network that is preferentially active during rest and deactivated during task engagement [91]. Altered functional integrity of the DMN has been demonstrated in several neuropsychiatric disorders, including TS. A study reported that functional connectivity in the DMN correlated negatively with tic severity in a subgroup of TS-pure tic patients [92]. It has also been reported that tic severity correlated negatively with abnormal intrinsic functional connectivity (iFC) between the bilateral anterior cingulate cortices [93]. The latter finding is in line with the hypothesis that impaired inter-hemispheric functional connectivity contributes to the pathophysiology of TS. This finding also suggests that the iFC could serve as a quantitative biomarker for clinical diagnosis. However, independent replication is required before this result can be well accepted.

In another study, the functional connectivity profile of TS patients who showed a good clinical response to CM-Pf DBS were compared with that of patients who displayed a poor clinical response [94]. The functional profile was defined in terms of connection between the volumes of tissue activated (VTAs) of the active DBS contact and the cortical areas. The results showed that responders had VTAs that were closely linked to the right frontal middle gyrus, the left frontal superior sulci region, and the left cingulate sulci region, whereas poor responders had VTAs that were only loosely related to these regions [94]. Although this study was limited by small sample size (*n* = 5 patients), the results indicate that the assessment of VTA-based functional connectivity profiles could help in predicting the patient’s clinical response to CM-Pf DBS.

In conclusion, the assessment of functional connectivity profiles seems to be a promising approach to identifying diagnostic or prognostic markers in TS. Functional effectivity profile assessment may similarly be useful in improving clinical outcome following STN–DBS in Parkinson’s disease [95]. It has also been postulated that long-term DBS can restore brain functional connectivity at a global level [96]. Accordingly, an important topic that warrants further research is the relationship between preoperative functional connectivity profiles and clinical outcomes in TS.

### Structural connectivity profiles

Neuroimaging studies have also assessed the structural connections in the human brain, usually employing regional measures. In a study, probabilistic stimulation atlases were used to identify anatomical regions that may predict the therapeutic response to DBS for TS [42]. However, the stimulation location relative to structural anatomy alone did not sufficiently predict the efficacy of DBS on tic severity. This study, however, focused on a single focal brain site. As brain regions are not isolated structures and the connectivity between regions is crucial for normal brain function, there has been a recent shift to methods that study the connectivity between regions. For example, tractography based on diffusion tensor imaging (DTI) can be used to identify the probabilistic structural connectivity of the site of stimulation and to detect the brain networks that contribute to symptom improvement across multiple surgical targets [97]. Also, DTI studies have shown altered properties of white matter microstructure in cortico-striato-thalamo-cortical circuitry in patients with TS [98, 99]. In another study, a large sample of young patients (age range, 8–21) were measured using tract-based spatial statistics and probabilistic tractography [100]. The results demonstrated both marked and wide spread decreases in axial diffusivity together with altered white matter connectivity. The tic severity was associated with increased connectivity between primary motor cortex and the caudate nuclei [100]. These results provide putative evidence that altered connectivity of the insula might play a pivotal role in the pathogenesis of TS.

Tractography has been used to analyze the network effects of DBS for treatment-refractory OCD patients [101, 102], demonstrating that optimal therapeutic results are associated with the activation of specific fiber pathways. In OCD DBS targeting NAc/ALIC, the degree of connectivity between stimulation sites and medial and lateral prefrontal cortices significantly predicted clinical improvement [102]. These results also indicate that connectivity of the site stimulation plays a role in mediating the clinical response to DBS. Selection and refinement of DBS targets based on structural connectivity by tractography could help in improving clinical outcomes and avoiding stimulation related adverse events of DBS therapy for TS.

As discussed before, DBS of the Forel’s field seems to be effective for tics and comorbid symptoms in TS, but the exact location of this target cannot be easily be estimated from the anatomical information provided by standard MRI and CT scans. Its reference coordinates obtained from stereotactic brain atlases or target using the surrounding structures as landmarks [51]. Direct targeting of the Forel’s field and their connective fiber tracts might be achieved using tractography guided approaches. Likewise, the superolateral branch of the medial forebrain bundle (slMFB) seems to be anatomically and functionally connected with DBS targets used to treat major depressive disorder (MDD), such as the NAc/ALIC [103]. In another study, tractography was useful for localizing and implanting DBS targeting the slMFB, serving to modulate subcortical and cortical reward-related pathways assumed to be dysfunctional in MDD [104]. The results showed, indeed, that direct white-matter modulation of slMFB fibers achieved desirable anti-depressive effects. Moreover, a double-blinded, randomized study involving 34 patients with either tremor-dominant Parkinson’s disease or essential tremor demonstrated the clinical utility of tractography. The results demonstrated that the tractography-guided lead placement produced a more enduring tremor control and fewer adverse effects compared with lead placement using conventional landmarks [105]. It also seems that tractography is feasible and effective in identifying the optimal DBS trajectory [106]. Surgeons can perform DBS procedures using the anatomical information from preoperative DTI studies for accurate DBS implantation.

## Conclusions

Tourette syndrome (TS) is a childhood-onset neuropsychiatric disorder characterized by the presence of multiple motor and vocal tics. TS usually co-occurs with one or more psychiatric disorders. Although behavioral and pharmacological treatments for TS are available, some patients do not profit from these treatments and continue to display significant and disabling symptoms. For severe and refractory cases of TS, DBS could provide an alternative treatment option. Important issues involved in DBS treatment include patient selection, clinical assessment including psychiatric comorbidities, selection of clinical outcomes, assessment of patient risks and benefits, DBS target selection, and treatment optimization. Recent recommendations for TS DBS have been published by the TAA and the AAN.

DBS seems to offer a valuable treatment option for severe and refractory cases of TS. Although several effective targets have been identified, different targets are associated with different therapeutic effects and different adverse-event profiles. However, the clinically best target or combination of targets remains to be determined. Multiple targets and/or DBS combined with radiosurgery are both promising approaches to improve clinical outcomes in carefully selected patients with severe psychiatric comorbidities. Individual patient differences in clinical response to TS DBS have been substantial, and a marker that can predict individual response has not yet been identified. In DBS of pediatric patients, clinicians are faced with various ethical issues, which need to be carefully considered on a case by case basis. The effect of conventional open-loop DBS on TS symptoms appears clinically significant, but newly developed, closed-loop DBS (aDBS) could greatly advance treatment for TS by adjusting in real-time the stimulation according to the clinical state of the patient and the underlying pathological network activity.

Finally, TS DBS should not be performed without an experienced multidisciplinary team, including a psychiatrist for pre- and post-operative clinical assessments. It is necessary to confirm the DSM-V diagnosis, to rule out psychogenic tics, and to assess psychiatric comorbidities. Age is not an eligibility criterion for DBS, but a multidisciplinary board should evaluate risks and benefits for each patient while taking into consideration the ethical issues relevant to pediatric populations.

## Data Availability

All data generated or analyzed during this study are included in this published article.

## Abbreviations

aDBS: Adaptive DBS
ADHD: Attention deficit hyperactivity disorder
ALIC: Anterior limb of internal capsule
amGPi: Anteromedial or limbic GPi
BAI: Beck Anxiety Inventory
BDI: Beck Depression Inventory
CGI-SI: Clinical Global Impression–Severity of Illness scale score
CM-Pf: Centromedian-parafascicular complex
DAPP-BQ: Dimensional Assessment of Personality Pathology–Basic Questionnaire
DBS: Deep brain stimulation
DMN: Default mode network
DTI: Diffusion tensor imaging
fMRI: Functional Magnetic Resonance Imaging
GAF: Global Assessment of Functioning Scale
GPi, GPe: Globus pallidus internal and external segments
HAMD: Hamilton Depression Scale
ICD: Impulse control disorder
iFC: Intrinsic functional connectivity
IPGs: Implantable pulse generators
LFP: Local field potential
MDD: Major depression
MRTRS: Modified Rush Tic Rating Scale
NAc: Nucleus accumbens
OCD: Obsessive-compulsive disorder
PD: Parkinson disease
QIDS-SR16: Quick Inventory Depression Scale – Self Report 16
QoL: Quality of life
SCL90: Symptoms Checklist List 90
slMFB: Superolateral branch of the medial forebrain bundle
STAI: State-Trait Anxiety Inventory
STN: Subthalamus nucleus
TAA: Tourette Association of America
TS: Tourette syndrome
VC: Ventral capsule
VS: Ventral striatum
VTA: Volume of tissue activated
Y-BOCS: Yale-Brown Obsessive Compulsive Scale
YGTSS: Yale Global Tic Severity Scale
